# Editorial

**DOI:** 10.1007/BF01146452

**Published:** 1992-03

**Authors:** Paul Goddard


### Editorial

As the new editor I would firstly like to record a debt of gratitude to Mike Wilson and his stirling efforts over the last few years. During his period as editor we have seen the standing of the journal rise so that it is now truly a journal that represents and reflects the views, news and reviews of doctors throughout the West Country. His editorial skills are thankfully not completely lost to the journal since he will be staying on as an assistant editor ..... we will effectively be swapping roles.

Without Mike's continuous efforts the journal would have disappeared long ago. Instead it has survived and grown. The quality of contributions has never been higher and we hope that the mix of articles provides a fascinating read and helps to educate and inform. I will endeavour to follow in the master's footsteps.

The only blight on the horizon is the problem of funding. Nuffield have very kindly supported the journal over the last three years and indeed are supporting the Spring and Summer issues this year. For this we are very grateful. From then on we are on our own. Watch this space for news.

Paul Goddard

